# Genetic Polymorphisms of Pharmacogenes among the Genetically Isolated Circassian Subpopulation from Jordan

**DOI:** 10.3390/jpm10010002

**Published:** 2020-01-06

**Authors:** Laith N. AL-Eitan, Doaa M. Rababa’h, Nancy M. Hakooz, Mansour A. Alghamdi, Rana B. Dajani

**Affiliations:** 1 Department of Applied Biological Sciences, Jordan University of Science and Technology, P.O. Box 3030, Irbid 22110, Jordan; doaa.mohammad89@yahoo.com; 2 Department of Biotechnology and Genetic Engineering, Jordan University of Science and Technology, Irbid 22110, Jordan; 3 Department of Biopharmaceutics and Clinical Pharmacy, School of Pharmacy, University of Jordan, Amman 11942, Jordan; nhakooz@ju.edu.jo; 4 College of Medicine, King Khalid University, Abha 61421, Saudi Arabia; m.alghamdi@kku.edu.sa; 5 Department of Biology and Biotechnology, Hashemite University, Zarqa 13133, Jordan; rdajani@hu.edu.jo; 6 Radcliffe Institute for Advanced Studies, Harvard University, Cambridge, MA 02138, USA

**Keywords:** pharmacogenomics, VIP polymorphism, phase I enzymes, phase II enzymes, metabolizing

## Abstract

Several genetic variants have been identified that cause variation among different populations and even within individuals of a similar descent. This leads to interindividual variations in the optimal dose of the drug that is required to sustain the treatment efficiency. In this study, 56 single nucleotide polymorphisms (SNPs) within several pharmacogenes were analyzed in 128 unrelated subjects from a genetically isolated group of Circassian people living in Jordan. We also compared these variant distributions to other ethnic groups that are available at two databases (Genome 1000 and eXAC). Our results revealed that the distribution of allele frequencies within genes among Circassians in Jordan showed similarities and disparities when compared to other populations. This study provides a powerful base for clinically relevant SNPs to enhance medical research and future pharmacogenomic studies. Rare variants detected in isolated populations can significantly guide to novel loci involved in the development of clinically relevant traits.

## 1. Introduction

The unique diversity among individuals and populations is because of the genetic variation in their genomes [1,2]. Several different genetic variants have been identified that cause variation among different populations and individuals of similar descent [3].

Single base pair substitution or transversion is called single nucleotide polymorphisms (SNP) [4,5,6]. SNP is the most common variant that occurs approximately in 1000 nucleotides. However to be considered a rare allele, its frequency must be at least 1% in a random sample of one population [7].

Approximately, over 7 million common SNPs have been identified worldwide [8]. The impact of SNPs on altering enzyme activity consequently affects the drug-metabolizing process [9,10] among individuals. Even though individuals share 99.6–99.8% of DNA sequences, 0.2–0.4% of variation are responsible for distinguishing differences among the different population and ethnic groups [11,12]. As a result, metabolic capacity varies among individuals. This fact provides the basis for an attempt to the enhancement of individual responses to medication by targeting the variants within patients’ genome [13]. Each individual respond, distribute, excrete and eliminate drugs in different ways [8].

This diversity is due to many parameters such as environmental factors (smoking and alcohol consumption) and genetic features including polymorphisms in genes that encode for cytochrome p450 oxidase (drug-metabolizing enzymes), drug targets (such as receptors) and drug transporter (ATP-binding cassette (ABC) transporters and solute carrier (SLC) transporters) [14]. These very important pharmacogenetic (VIP) variants are 126 variants in 44 different gene coding enzymes (PharmGKB: http://www.pharmgkb.org) [15,16]. Cytochrome P450 (*CYP450*) enzymes are important for cholesterol and steroid production. In addition, they are essential for the detoxification of exogenous chemicals and drug metabolism. More than 50 CYP450 enzymes have been identified, however, the CYP1A2, CYP2C9, CYP2C19, CYP2D6, CYP3A4 and CYP3A5 are the main drugs metabolizing enzymes [17].

The VIP variants of *CYP450* genes have been implicated in inter-individual variability in drug response and adverse drug reactions [18]. Phase II drug metabolizing enzymes are detoxifying enzymes that undergo conjugating reactions and play a role in converting xenobiotics and endogenous substances into easily removable forms [19]. In addition, they serve in the metabolic inactivation of active pharmacological substrates. The conjugation of Phase II biotransformation reactions includes glucuronidation, methylation, acetylation, sulfation, glutathione and amino acid conjugation [20]. Phase II drug-metabolizing transferases comprise sulfotransferases (SULTs), UDP-glucuronosyltransferases (UGTs), N-acetyltransferases (NATs), glutathione S-transferases (GSTs) and various methyltransferases (thiopurine S-methyl transferase (TPMT) and catechol O-methyl transferase (COMT)) [21].

The metabolic rate can be influenced by genetic variants within a specific gene, for example; ADH (alcohol dehydrogenase) enzymes convert alcohol to acetaldehyde and acetaldehyde to acetate, which is influenced by functional polymorphisms within *ADH1B*, and this may increase the prevalence of alcoholic liver diseases [22]. In addition, many significant variants have been notified within the *SLC19A1* gene from solute carrier family and were clinically functional including; rs1051266, rs12659 and rs1131596 [23].

In this study, we aimed to determine the allele frequencies of clinically relevant VIP SNPs within several pharmacogenes the Circassian living in Jordan in comparison to other ethnic groups.

## 2. Materials and Methods

### 2.1. Study Subjects

This study involved unrelated subjects from the isolated group (128 Circassians) who live in Jordan. Of venous blood 10 mL was collected from each participant for molecular analyses. This study was conducted in agreement with the human ethics committee at the National Center for Diabetes, Endocrinology and Genetics (NCDEG) and Jordan University of Science and Technology (JUST). Written informed consent was obtained from all volunteers in the study.

### 2.2. DNA Extraction and Genotyping

Genomic DNA was extracted from each blood sample using the Wizard^®^ Genomic DNA Purification Kit (Promega Corporation, Madison, WI, USA) according to the manufacturer’s instructions. The quality and quantity of the purified DNA was ascertained via agarose gel electrophoresis and the Nano-Drop ND-1000 UV-Vis Spectrophotometer (Thermo Scientific, Wilmington, DE, USA), respectively.

Candidate genes variants were analyzed using the sequencing technique. Multiplex PCR was used to amplify loci of candidate SNPs followed by a primer extention process (Mass EXTEND) resulting in allele-specific DNA products. Mass spectrometry was used for minisequencing reaction product analysis. Afterward, the extension PCR products were separated onto a 384 well spectroCHIP and placed into the MALDI-TOF (matrix assisted laser desorption/ionization time-of-flight) mass spectrometer. Finally, a software system (SpectroTYPER-RT (RT for real-time) was used to analyze the results.

### 2.3. Populations Variation Data

The allele counts variation data for other populations were obtained from HapMap Project website (https://www.ncbi.nlm.nih.gov/variation/tools/1000genomes/): CHE: Chechens from Jordan, ASW: African ancestry in Southwest USA, CEU: Utah, USA residents with Northern and Western European ancestry from the CEPH collection, CHB: Han Chinese in Beijing, China, CDX: Chinese Dai in Xishuangbanna, China, GIH: Gujarati Indians in Houston, Texas, USA, GBR: British in England and Scotland, JPT: Japanese in Tokyo, Japan, LWK: Luhya in Webuye, Kenya, MXL: Mexican ancestry in Los Angeles, California, USA, TSI: Toscani in Italy, YRI: Yoruba in Ibadan, Nigeria, CAR: Circassian from Jordan (unpublished data) and ACB; African Caribbean in Barbados. In addition, the allele count data of populations around the world were obtained from the Exome Aggregation Consortium (ExAC) at http://exac.broadinstitute.org: African, East Asian, Latino, European (Non-Finnish), South Asian and European (Finnish).

### 2.4. Statistical Analysis

Statistical analyses were conducted using the Statistical Package for Social Sciences SPSS (version 19). The genotype and allele frequency was calculated and tested by performing the Hardy–Weinberg equilibrium equation (HWE) and all *p*-values accepted as *p*-value < 0.05.

## 3. Results

### 3.1. Basic Characteristics of the Polymorphisms of the Candidate Genes

Table 1 shows the candidate SNPs within 28 genes in addition to the quality control of genotyping with genotype call rates ranging from 96.5% to 100%. Other data about the included SNPs such as the chromosomal position, minor allele and location are illustrated in Table 1.

### 3.2. Basic Characteristics of Selected Variants within the Pharmacogenes with Their Allele and Genotype Frequencies

Allele and genotype frequencies of the selected variants among 128 Circassians are listed in Table 2. In addition, polymorphisms were tested for Hardy–Weinberg Equilibrium (HWE), SNPs that did not fulfill the HWE equation (*p*-value < 0.05) were excluded from this study.

### 3.3. Significant Genetic Variants in Circassian Compared to Other Populations

Table 3 compares between minor allele frequencies (MAF) of significant polymorphisms among Circassians and other populations available at genome 1000. For example; rs1805124 was distributed among Circassian significantly different from CHB, CDX and JPT populations but similar to it within ASW, GBR and TSI. In addition, Table 3 compare the allelic distribution of selected variants among the Circassians and other populations by estimating the *p*-value. For instance, we found that the *CYP2J2* variant (rs890293) distribution within Circassian population in Jordan was different from ASW, CDX, JPT and LWK populations.

Furthermore, the frequencies of significant variants within the selected genes among Circassian were compared to the available frequency variants among six populations listed in the Exome Aggregation Consortium (ExAC) database Table 4. Our results revealed that several variants among Circassians in Jordan had significantly different frequencies compared to other populations.

## 4. Discussion

An isolated population emerges when a small number of individuals are isolated from their founder group and establish a new population. The homogeneity of the isolated populations with respect to environmental and cultural aspects render them to be an ideal choice for various studies in order to exclude the impact of environmental factors on disease evolution [24,25]. The genetic etiology of complex diseases can be studied by identifying rare genetic variants. Therefore, genetically isolated populations have been a strong resource for novel variant discovery [25]. In this study, we analyzed many significant SNPs of critical genes within the isolated Circassian group in Jordan. Variants were chosen based on their clinical significance. These variants were also selected from pervious published polymorphisms within different pharmacogenes undergoing important roles in drug metabolism.

Moreover, we compared the distribution of these variants among Circassians to its other populations. However, our findings showed disparities and similarities. Remarkably, among Circassian, the frequency of the globally identified minor allele (C) of rs3807375 within *KCNH2* gene was 0.68, which is different from other frequencies among most of the other populations. This significant variant genotypes (TT and CT) were found in association with increased QT interval as compared to (CC) genotype [26]. Moreover, we noticed that the distribution of both polymorphisms rs1131596 and rs1051266 of *SLC19A1* gene were the same among populations. However, allele C of rs1051266 was not associated with pediatric ALL therapy outcome [27]. The variation in the allele frequencies between Circassians and other populations can be attributed to many factors such as genetic isolation, migration (gene flow), mutation and natural selection [28].

Within Phase I drug-metabolizing genes, in particular, cytochrome 450 enzymes there are many genetic variants termed VIP variants that have been shown to be associated with different diseases. These variants are important for pharmacogenetics. However, individuals show the variable activity of drug metabolism depending on the genetic allele of *CYP450* genes. The Metabolizing activity of individuals is classified into three groups, poor metabolizers when individuals have two copies of the variant alleles; then there are those with heterozygote alleles (wild-type and variant) who have reduced enzyme activity. Other individuals who inherit multiple copies of wild-type alleles are ultrarapid metabolizers, which result in enzyme hyperactivity [28]. It has been reported that 7% of white individuals are poor metabolizers of drugs metabolized by CYP2D6. On the other hand, 20% of Asians are poor metabolizers of drugs dependent on CYP2C19 [17].

On the other hand, many genetic polymorphisms within Phase II drug metabolizing genes were defined as VIP variants and related to different diseases important to pharmacogenetics. Within *UGT1A1* gene the rs8175347 and rs10929302 (AA) have been implicated in toxicity, increased risk of diarrhea and neutropenia when treated with irinotecan in people with colorectal neoplasms [29,30]. A functional single nucleotide polymorphism of *COMT* gene (rs4680) has been studied and results in the substitution of valine for methionine leading to reduced activity of the enzyme. This significant variant has been associated with a number of neurological disorders such as schizophrenia and Parkinson’s disease [31,32].

This study mainly concentrated on defining alleles frequencies of different pharmacogenes within an isolated population (Circassian) living in Jordan. However, this study encountered some limitations such as small sample size and focusing on genotyping certain polymorphisms. Moreover, only a few pharmacogenetic studies [33,34,35,36,37,38,39,40,41,42,43,44] have been conducted in Jordan. Therefore, more studies are recommended in this topic by investigating other VIP variants in different candidate genes. The relationship between rare variants of an isolated population and complex diseases should be also investigated to identify which variants are associated with susceptibility to different genetic diseases and responsiveness to treatment. Further investigations including a larger sample size are also required to validate the presented results to this minority group from Jordan.

In summary, this study shows that the large level of genetic variability between populations can serve as a fundamental resource for the worldwide distribution of allele frequencies that is important for the future of pharmacogenomics and personalized medicine. Finally, identification of the genotypic and allelic distributions and frequencies of VIP variants in such a minority group helps in providing a clinical basis for safer drug administration, better therapeutic effects and different treatment options.

## Figures and Tables

**Table 1:** List of genes, their single nucleotide polymorphisms (SNPs), positions and genotyping data for Circassian (*N* = 128).

Gene	SNP_ID	Position ^a^	SNP	SNP Location	Assay Pass Rate ^b^	Call Rate ^c^
***MTHFR***	rs1801131	1:11794419	A > C^MA^	Missense variant	100%	100%
	rs1801133	1:11796321	C > T^MA^	Missense variant	100%	99.7%
***DPYD***	rs3918290	1:97450058	C > T^MA^	Splice Donor variant	100%	99.7%
***PTGS2***	rs689466	1:186681619	G > A^MA^	2KB Upstream variant	100%	100%
***SCN5A***	rs7626962	3:38579416	G > A/G>T	Missense variant	100%	100%
	rs1805124	3:38603929	T > C^MA^	Missense variant	100%	100%
	rs6791924	3:38633208	G > A^MA^	Missense variant	100%	100%
***NR1I2***	rs3814055	3:119781188	C > T^MA^	5 Prime UTR variant	100%	100%
***P2RY12***	rs2046934	3:151339854	G^MA^ > A	Intron variant	99%	99.2%
***P2RY1***	rs1065776	3:152835839	C > T^MA^	Synonymous variant	100%	100%
	rs701265	3:152836568	A > G^MA^	Synonymous variant	100%	100%
***ADH1A***	rs975833	4:99280582	G > C^MA^	Intron variant	100%	99.7%
***ADH1B***	rs2066702	4:99307860	G > A^MA^	Missense variant	100%	100%
	rs1229984	4:99318162	T^MA^ > C	Missense variant	100%	100%
***ADH1C***	rs698	4:99339632	T > C^MA^	Missense variant	99%	99.2%
***HMGCR***	rs17244841	5:75347030	A > T^MA^	Intron variant	99%	98.6%
	rs3846662	5:75355259	A^MA^ > G	Intron variant	100%	99.7%
	rs17238540	5:75359673	T > G^MA^	Intron variant	100%	100%
***ADRB2***	rs1042713	5:148826877	G > A^MA^	Missense variant	100%	100%
	rs1042714	5:148826910	G^MA^ > C	Stop gained	100%	100%
	rs1800888	5:148827322	C > T^MA^	Missense variant	100%	99.7%
***AHR***	rs2066853	7:17339486	G > A^MA^	Missense variant	100%	99.7%
***KCNH2***	rs3815459	7:150947306	C > T^MA^	Intron variant	100%	99.7%
	rs3807375	7:150970122	C > T^MA^	Intron variant	100%	100%
***KCNJ11***	rs5219	11:17388025	T^MA^ > C	Stop gained	100%	100%
***SLCO1B1***	rs4149056	12:21178615	T > C^MA^	Missense variant	100%	100%
***VKORC1***	rs7294	16:31091000	C > T^MA^	3 Prime UTR variant	100%	100%
	rs9934438	16:31093557	G > A^MA^	Intron variant	100%	100%
***SLC19A1***	rs12659	21:45531642	A^MA^ > G	Synonymous variant	100%	100%
	rs1051266	21:45537880	T > C^MA^	Missense variant	99%	98.6%
	rs1131596	21:45538002	G > A^MA^	Missense variant	100%	99.7%
***CYP2J2***	rs890293	1:59926822	C > A^MA^	Upstream variant	100%	100%
***CYP3A4***	rs4986913	7:99760836	G > A^MA^	Missense variant	100%	100%
	rs4986910	7:99760901	A > G^MA^	Missense variant	100%	100%
	rs4986909	7:99762047	G > A ^MA^	Missense variant	100%	100%
	rs12721634	7:99784038	A > G	Missense variant	100%	100%
	rs2740574	7:99784473	C^MA^ > T	Upstream variant	100%	100%
***CYP2C19***	rs4986893	10:94780653	G > A^MA^	Stop gained	100%	100%
	rs4244285	10:94781859	G > A^MA^	Synonymous variant	100%	99.7%
***CYP2C9***	rs1799853	10:94942290	C > T^MA^	Missense variant	100%	100%
***CYP2A6***	rs28399454	19:40845362	C > T^MA^	Missense variant	100%	100%
	rs1801272	19:40848628	A > T^MA^	Missense variant	100%	100%
	rs28399433	19:40850474	A > C^MA^	Upstream variant	99%	99.2%
***CYP2B6***	rs3745274	19:41006936	G > T^MA^	Missense variant	96%	96.5%
	rs28399499	19:41012316	T > C^MA^	Missense variant	100%	100%
***CYP2D6***	rs59421388	22:42127608	C > T^MA^	Missense variant	100%	100%
	rs28371725	22:42127803	C > T^MA^	Intron variant	98%	98.4%
	rs61736512	22:42129132	C > T^MA^	Missense variant	100%	100%
	rs28371706	22:42129770	G > A^MA^	Missense variant	100%	100%
	rs5030656	22: 42128174	delTCT	Inframe deletion	100%	100%
***UGT1A1***	rs4124874	2:233757013	T^MA^ > G	Intronic variant	100%	100%
	rs10929302	2:23375713	G > A^MA^	Intronic variant	100%	100%
	rs4148323	2:233760498	G > A^MA^	Missense variant	100%	100%
***COMT***	rs4680	22:19963748	G > A^MA^	Missense variant	100%	99.7%
***GSTP1***	rs1695	11:67585218	A > G^MA^	Missense variant	100%	100%
	rs1138272	11:67586108	C > T^MA^	Missense variant	100%	100%

a. Chromosome positions are based on NCBI Human Genome Assembly Build. b. Ratio of the number of discordant genotypes to the number of duplicates. c. Ratio of the number of valid genotypes to the number of subjects genotyped. MA: global minor allele.

**Table 2:** The minor allele frequencies and HWE *p* values for genes variants in Circassian (*N* = 128).

Gene	SNP_ID	Alleles	Allelic Frequency	Genotypes	Genotypic Frequency	*p*-Value *
***MTHFR***	rs1801131	C A	0.35 0.65	AA CA CC	0.42 0.48 0.11	0.7
	rs1801133	T C	0.33 0.67	CC CT TT	0.42 0.49 0.08	0.24
***DPYD***	rs3918290	C	1.0	CC	1.0	NA
***PTGS2***	rs689466	T C	0.83 0.17	CC CT TT	0.02 0.3 0.68	0.52
***SCN5A***	rs7626962	G	1.0	GG	1.0	NA
	rs1805124	T C	0.75 0.25	CC CT TT	0.07 0.37 0.56	0.82
	rs6791924	G	1.0	GG	1.0	NA
***NR1I2***	rs3814055	C T	0.53 0.47	CC CT TT	0.28 0.5 0.22	0.95
***P2RY12***	rs2046934	A G	0.93 0.07	AA AG	0.87 0.13	0.42
***P2RY1***	rs1065776	C T	0.98 0.02	CC CT	0.96 0.04	0.82
	rs701265	A G	0.86 0.14	AA AG GG	0.74 0.25 0.02	0.71
***ADH1A***	rs975833	G C	0.63 0.37	CC CG GG	0.11 0.53 0.36	0.19
***ADH1B***	rs2066702	G A	0.97 0.03	GA GG	0.05 0.95	0.75
	rs1229984	C T	0.87 0.13	CC CT TT	0.75 0.24 0.01	0.69
***ADH1C***	rs698	C T	0.25 0.75	CC CT TT	0.05 0.41 0.54	0.36
***HMGCR***	rs17244841	A T	0.98 0.02	AA AT	0.97 0.03	0.85
	rs3846662	G A	0.52 0.48	AA GA GG	0.25 0.47 0.29	0.48
	rs17238540	G T	0.02 0.98	TT GT	0.97 0.03	0.86
***ADH1C***	rs698	T C	0.75 0.25	TT TC CC	0.05 0.41 0.54	0.36
***ADRB2***	rs1042713	G A	0.54 0.46	GG GA AA	0.25 0.58 0.17	0.079
	rs1042714	C G	0.68 0.32	CC CG GG	0.44 0.48 0.08	0.23
	rs1800888	C	1.0	CC	1.0	N/A
***AHR***	rs2066853	G A	0.84 0.16	GG GA AA	0.72 0.24 0.04	0.31
***KCNH2***	rs3815459	C T	0.73 0.27	CC CT TT	0.52 0.43 0.05	0.37
	rs3807375	C T	0.68 0.32	CC CT TT	0.47 0.43 0.1	0.97
***KCNJ11***	rs5219	C T	0.59 0.41	CC CT TT	0.35 0.48 0.17	0.86
***SLCO1B1***	rs4149056	C T	0.16 0.84	CC CT TT	0.01 0.31 0.68	0.19
***VKORC1***	rs7294	C T	0.67 0.33	CC CT TT	0.43 0.47 0.1	0.69
	rs9934438	G A	0.53 0.47	GG GA AA	0.22 0.52 0.27	0.86
***SLC19A1***	rs12659	G A	0.55 0.45	GG GA AA	0.29 0.52 0.18	0.6
	rs1051266	C T	0.58 0.42	CC CT TT	0.3 0.55 0.15	0.15
	rs1131596	A G	0.58 0.42	AA AG GG	0.3 0.55 0.15	0.15
***CYP2J2***	rs890293	C A	0.92 0.08	AA CA CC	0.01 0.14 0.85	0.55
***CYP3A5***	rs10264272	C	1.0	CC	1.0	N/A
	rs776746	C T	0.91 0.09	CC CT	0.82 0.18	0.6
***CYP3A4***	rs4986913	G	1.0	GG	1.0	N/A
	rs4986910	A G	0.99 0.01	AA AG	0.98 0.02	0.89
	rs4986909	G	1.0	GG	1.0	N/A
	rs12721634	A	1.0	AA	1.0	N/A
	rs2740574	T C	0.97 0.03	TC TT	0.05 0.95	0.75
***CYP2C19***	rs4986893	G A	0.004 0.99	GG GA	0.99 0.01	0.96
	rs4244285	G A	0.92 0.08	AA GA GG	0.01 0.14 0.85	0.56
***CYP2C9***	rs1799853	C T	0.88 0.12	CC CT TT	0.79 0.19 0.02	0.38
***CYP2A6***	rs28399454	C	1.0	CC	1.0	N/A
	rs1801272	A	1.0	AA	1.0	N/A
	rs28399433	A C	0.93 0.07	AA AC	0.86 0.14	0.39
***CYP2B6***	rs3745274	G T	0.8 0.2	GG GT TT	0.62 0.36 0.02	0.40
	rs28399499	T	1.0	TT	1.0	N/A
***CYP2D6***	rs59421388	C	1.0	CC	1.0	N/A
	rs28371725	C T	0.85 0.15	CC CT TT	0.73 0.25 0.02	0.73
	rs61736512	C	1.0	CC	1.0	N/A
	rs28371706	G A	0.99 0.01	GG GA	0.98 0.02	0.92
	rs5030656	C/T/T	1.0	-	1.0	N/A
***UGT1A1***	rs4124874	G T	0.54 0.46	GG GT TT	0.33 0.26 0.41	0.06
	rs10929302	G A	0.61 0.39	GG GA AA	0.37 0.47 0.16	0.90
	rs4148323	G A	0.97 0.03	GG GA	0.95 0.05	0.80
***COMT***	rs4680	G A	0.59 0.41	GG GA AA	0.35 0.47 0.18	0.90
***GSTP1***	rs1695	A G	0.67 0.33	AA AG GG	0.46 0.12 0.43	0.69
	rs1138272	C T	0.84 0.16	CC CT TT	0.71 0.26 0.02	0.50

* *p*-value > 0.05 is considered normally distributed, N/A: not applicable.

**Table 3:** Significant variants within several genes in Circassian compared to HapMap populations.

SNP ID	Populations												
	CAR	ASW	CEU	CHB	CDX	GBR	GIH	JPT	LWK	MXL	TSI	YRI	ACB
rs689466	0.17 ^a^	0.13 ^a^	0.19 ^a^	0.47 ^a^	0.52 ^a^	0.20 ^a^	0.13 ^a^	0.44 ^a^	0.03 ^a^	0.26 ^a^	0.20 ^a^	0.07 ^a^	0.10 ^a^
		0.7 ^b^	0.5 ^b^	N/A ^b^	N/A ^b^	0.7 ^b^	1.4 ^b^	N/A ^b^	21.4 ^b^	4.6 ^b^	0.6 ^b^	9.1 ^b^	3.5 ^b^
		0.38 ^c^	0.46 ^c^	N/A ^c^	N/A ^c^	0.38 ^c^	0.23 ^c^	N/A ^c^	4 × 10^−7 c^	0.03 ^c^	0.44 ^c^	3 × 10^−3 c^	0.06 ^c^
rs1805124	0.25 ^a^	0.26 ^a^	0.18 ^a^	0.12 ^a^	0.07 ^a^	0.22 ^a^	0.20 ^a^	0.13 ^a^	0.30 ^a^	0.16 ^a^	0.23 ^a^	0.32 ^a^	0.28 ^a^
		0.03 ^b^	3.4 ^b^	11.8 ^b^	19.7 ^b^	0.5 ^b^	1.6 ^b^	10.2 ^b^	1.4 ^b^	4.0 ^b^	0.26 ^b^	3.2 ^b^	0.4 ^b^
		0.86 ^c^	0.07 ^c^	1 × 10^−3 c^	3 × 10^−5 c^	0.49 ^c^	0.20 ^c^	1 × 10^−3 c^	0.24 ^c^	0.05 ^c^	0.61 ^c^	0.07 ^c^	0.51 ^c^
rs3814055	0.47 ^a^	0.29 ^a^	0.34 ^a^	0.27 ^a^	0.15 ^a^	0.40 ^a^	0.42 ^a^	0.25 ^a^	0.30 ^a^	0.33 ^a^	0.36 ^a^	0.27 ^a^	0.39 ^a^
		10.0 ^b^	7.4 ^b^	18.2 ^b^	N/A ^b^	1.8 ^b^	1.1 ^b^	21.9 ^b^	12.3 ^b^	5.9 ^b^	5.3 ^b^	18.5 ^b^	4.3 ^b^
		2 × 10^−3 c^	0.01 ^c^	2 × 10^−5 c^	N/A ^c^	0.18 ^c^	0.30 ^c^	3 × 10^−6 c^	4 × 10^−4 c^	0.01 ^c^	0.02 ^c^	2 × 10^−5 c^	0.04 ^c^
rs1065776	0.02 ^a^	0.20 ^a^	0.05 ^a^	0.03 ^a^	0.02 ^a^	0.02 ^a^	0.10 ^a^	0.07 ^a^	0.21 ^a^	0.05 ^a^	0.03 ^a^	0.22 ^a^	0.20 ^a^
		N/A ^b^	3.4 ^b^	1.0 ^b^	0.1 ^b^	0.04 ^b^	14.9 ^b^	7.9 ^b^	N/A ^b^	2.4 ^b^	0.9 ^b^	N/A ^b^	N/A ^b^
		N/A ^c^	0.03 ^c^	0.32 ^c^	0.81 ^c^	0.84 ^c^	1 × 10^−4 c^	5 × 10^−3 c^	N/A ^c^	0.12 ^c^	0.35 ^c^	N/A ^c^	N/A ^c^
rs701265	0.14 ^a^	0.67 ^a^	0.17 ^a^	0.28 ^a^	0.19 ^a^	0.13 ^a^	0.21 ^a^	0.27 ^a^	0.80 ^a^	0.20 ^a^	0.16 ^a^	0.81 ^a^	0.72 ^a^
		N/A ^b^	0.70 ^b^	16.0 ^b^	2.0 ^b^	0.04 ^b^	4.0 ^b^	13.3 ^b^	N/A ^b^	2.7 ^b^	0.6 ^b^	N/A ^b^	N/A ^b^
		N/A ^c^	0.40 ^c^	1 × 10^−4 c^	0.15 ^c^	0.84 ^c^	0.04 ^c^	3 × 10^−4 c^	N/A ^c^	0.10 ^c^	0.44 ^c^	N/A ^c^	N/A ^c^
rs975833	0.37 ^a^	0.25 ^a^	0.24 ^a^	0.79 ^a^	0.75 ^a^	0.24 ^a^	0.49 ^a^	0.80 ^a^	0.19 ^a^	0.13 ^a^	0.27 ^a^	0.30 ^a^	0.28 ^a^
		5.2 ^b^	8.7 ^b^	N/A ^b^	N/A ^b^	8.3 ^b^	5.6 ^b^	N/A ^b^	17.6 ^b^	23.6 ^b^	5.4 ^b^	2.7 ^b^	4.6 ^b^
		0.02 ^c^	3 × 10^−3 c^	N/A ^c^	N/A ^c^	4 × 10^−3 c^	0.01 ^c^	N/A ^c^	3 × 10^−5 c^	1 × 10-^6 c^	0.02 ^c^	0.10 ^c^	0.03 ^c^
rs2066702	0.03 ^a^	0.20 ^a^	0.00 ^a^	0.00 ^a^	0.00 ^a^	0.00 ^a^	0.00 ^a^	0.00 ^a^	0.14 ^a^	0.03 ^a^	0.00 ^a^	0.28 ^a^	0.19 ^a^
		N/A ^b^	5.4 ^b^	5.6 ^b^	5.1 ^b^	5.0 ^b^	5.6 ^b^	5.6 ^b^	20.9 ^b^	0.1 ^b^	5.9 ^b^	N/A ^b^	N/A ^b^
		N/A ^c^	0.02 ^c^	0.02 ^c^	0.02 ^c^	0.03 ^c^	0.02 ^c^	0.02 ^c^	5 × 10^−6 c^	0.81 ^c^	0.01 ^c^	N/A ^c^	N/A ^c^
rs1229984	0.13 ^a^	0.00 ^a^	0.02 ^a^	0.71 ^a^	0.63 ^a^	0.01 ^a^	0.02 ^a^	0.73 ^a^	0.00 ^a^	0.08 ^a^	0.05 ^a^	0.00 ^a^	0.01 ^a^
		17.0 ^b^	19.4 ^b^	N/A ^b^	N/A ^b^	22.2 ^b^	16.2 ^b^	N/A ^b^	27.1 ^b^	1.4 ^b^	7.9 ^b^	29.4 ^b^	21.0 ^b^
		4 × 10^−5 c^	1 × 10^−5 c^	N/A ^c^	N/A ^c^	2 × 10^−6 c^	6 × 10^−5 c^	N/A ^c^	2 × 10^−7 c^	0.23 ^c^	5 × 10^−3 c^	6 × 10^−8 c^	5 × 10^−6 c^
rs698	0.25 ^a^	0.14 ^a^	0.47 ^a^	0.05 ^a^	0.11 ^a^	0.44 ^a^	0.28 ^a^	0.07 ^a^	0.14 ^a^	0.28 ^a^	0.31 ^a^	0.07 ^a^	0.11 ^a^
		6.4 ^b^	24.09 ^b^	N/A ^b^	13.7 ^b^	16.5 ^b^	0.4 ^b^	27.6 ^b^	8.7 ^b^	0.32 ^b^	1.7 ^b^	28.3 ^b^	14.8 ^b^
		0.01 ^c^	1 × 10^−6 c^	N/A ^c^	2 × 10^−4 c^	5 × 10^−5 c^	0.50 ^c^	25 × 10^−8 c^	3 × 10^−3 c^	0.56 ^c^	0.19 ^c^	1 × 10^−7 c^	1 × 10^−5 c^
rs17244841	0.02 ^a^	0.10 ^a^	0.01 ^a^	0.00 ^a^	0.00 ^a^	0.03 ^a^	0.00 ^a^	0.02 ^a^	0.08 ^a^	0.04 ^a^	0.03 ^a^	0.09 ^a^	0.10 ^a^
		14.1 ^b^	0.03 ^b^	3.2 ^b^	2.9 ^b^	0.76 ^b^	3.2 ^b^	0.1 ^b^	12.6 ^b^	2.1 ^b^	1.5 ^b^	14.5 ^b^	17.1 ^b^
		2 × 10^−4 c^	0.85 ^c^	0.07 ^c^	0.09 ^c^	0.38 ^c^	0.07 ^c^	0.75 ^c^	4 × 10^−4 c^	0.14 ^c^	0.21 ^c^	1 × 10^−4 c^	3 × 10^−5 c^
rs3846662	0.48 ^a^	0.13 ^a^	0.57 ^a^	0.48	0.49 ^a^	0.55 ^a^	0.34 ^a^	0.47 ^a^	0.03 ^a^	0.56 ^a^	0.57 ^a^	0.04 ^a^	0.11 ^a^
		N/A ^b^	4.1 ^b^	0.01 ^b^	0.1 ^b^	1.02 ^b^	9.3 ^b^	0.04 ^b^	N/A ^b^	2.3 ^b^	3.7 ^b^	N/A ^b^	N/A ^b^
		N/A ^c^	0.04 ^c^	0.91 ^c^	0.77 ^c^	0.15 ^c^	2 × 10^−3 c^	0.84 ^c^	N/A ^c^	0.13 ^c^	0.05 ^c^	N/A ^c^	N/A ^c^
rs17238540	0.02 ^a^	0.10 ^a^	0.01 ^a^	0.00 ^a^	0.00 ^a^	0.02 ^a^	0.00 ^a^	0.00 ^a^	0.09 ^a^	0.04 ^a^	0.03 ^a^	0.10 ^a^	0.09 ^a^
		14.2 ^b^	N/A ^b^	3.2 ^b^	2.9 ^b^	0.26 ^b^	3.2 ^b^	3.2 ^b^	14.0 ^b^	2.1 ^b^	1.5 ^b^	15.9 ^b^	14.6 ^b^
		2 × 10^−4 c^	N/A ^c^	0.07 ^c^	0.09 ^c^	0.61 ^c^	0.07 ^c^	0.07 ^c^	2 × 10^−4 c^	0.14 ^c^	0.21 ^c^	7 × 10^−5 c^	1 × 10^−4 c^
rs1042713	0.46 ^a^	0.54 ^a^	0.35 ^a^	0.55 ^a^	0.58 ^a^	0.42 ^a^	0.43 ^a^	0.44 ^a^	0.49 ^a^	0.48 ^a^	0.37 ^a^	0.53 ^a^	0.51 ^a^
		2.7 ^a^	5.5 ^b^	3.7 ^b^	6.5 ^b^	0.6 ^b^	0.31 ^b^	0.11 ^b^	0.46 ^b^	0.12 ^b^	3.7 ^b^	2.6 ^b^	1.2 ^b^
		0.09 ^c^	0.02 ^c^	0.05 ^c^	0.01 ^c^	0.40 ^c^	0.58 ^c^	0.74 ^c^	0.49 ^c^	0.72 ^c^	0.05 ^c^	0.10 ^c^	0.26 ^c^
rs1042714	0.32 ^a^	0.12 ^a^	0.46 ^a^	0.11 ^a^	0.10 ^a^	0.39 ^a^	0.23 ^a^	0.06 ^a^	0.21 ^a^	0.14 ^a^	0.14 ^a^	0.12 ^a^	0.16 ^a^
		16.8 ^b^	10.1 ^b^	29.7 ^b^	28.9 ^b^	2.7 ^b^	4.7 ^b^	N/A ^b^	6.5 ^b^	14.2 ^b^	2.7 ^b^	26.4 ^b^	14.6 ^b^
		4 × 10^−5 c^	1 × 10^−3 c^	5 × 10^−8 c^	7 × 10^−8 c^	0.09 ^c^	0.03 ^c^	N/A ^c^	0.01 ^c^	2 × 10^−4 c^	0.09 ^c^	21 × 10^−8 c^	1 × 10^−4 c^
rs2066853	0.16 ^a^	0.34 ^a^	0.09 ^a^	0.37 ^a^	0.26 ^a^	0.09 ^a^	0.12 ^a^	0.46 ^a^	0.48 ^a^	0.13 ^a^	0.09 ^a^	0.45 ^a^	0.47 ^a^
		16.6 ^b^	4.5 ^b^	29 ^b^	6.6 ^b^	3.3 ^b^	1.7 ^b^	N/A ^b^	N/A ^b^	0.45 ^b^	3.7 ^b^	N/A ^b^	N/A ^b^
		4 × 10^−5 c^	0.03 ^c^	7 × 10^−8 c^	0.01 ^c^	0.06 ^c^	0.19 ^c^	N/A ^c^	N/A ^c^	0.49 ^c^	0.05 ^c^	N/A ^c^	N/A ^c^
rs3815459	0.27 ^a^	0.42 ^a^	0.20 ^a^	0.71 ^a^	0.56 ^a^	0.15 ^a^	0.35 ^a^	0.80 ^a^	0.39 ^a^	0.39 ^a^	0.23 ^a^	0.33 ^a^	0.31 ^a^
		8.4 ^b^	3.2 ^b^	N/A ^b^	6.7 ^b^	8.3 ^b^	2.7 ^b^	N/A ^b^	7.4 ^b^	8.7 ^b^	0.79 ^b^	2.3 ^b^	1.2 ^b^
		3 × 10^−3 c^	0.07 ^c^	N/A ^c^	0.02 ^c^	4 × 10^−3 c^	0.09 ^c^	N/A ^c^	0.01 ^c^	3 × 10^−3 c^	0.37 ^c^	0.12 ^c^	0.26 ^c^
rs3807375	0.68 ^a^	0.27 ^a^	0.65 ^a^	0.25 ^a^	0.26 ^a^	0.70 ^a^	0.61 ^a^	0.20 ^a^	0.20 ^a^	0.43 ^a^	0.66 ^a^	0.23 ^a^	0.26 ^a^
		N/A ^b^	0.55 ^b^	N/A ^b^	N/A ^b^	0.17 ^b^	2.6 ^b^	N/A ^b^	N/A ^b^	N/A ^b^	0.35 ^b^	N/A ^b^	N/A ^b^
		N/A ^c^	0.45 ^c^	N/A ^c^	N/A ^c^	0.67 ^c^	0.10 ^c^	N/A ^c^	N/A ^c^	N/A ^c^	0.55 ^c^	N/A ^c^	N/A ^c^
rs5219	0.41 ^a^	0.14 ^a^	0.38 ^a^	0.38 ^a^	0.23 ^a^	0.26 ^a^	0.42 ^a^	0.33 ^a^	0.01 ^a^	0.41 ^a^	0.29 ^a^	0.00 ^a^	0.06 ^a^
		27.3 ^b^	0.26 ^b^	0.40 ^b^	16.1 ^b^	9.7 ^b^	0.04 ^b^	2.8 ^b^	N/A ^b^	1 × 10^−3 b^	7.7 ^b^	N/A ^b^	N/A ^b^
		17 × 10^−8 c^	0.60 ^c^	0.52 ^c^	1 × 10^−4 c^	2 × 10^−3 c^	0.83 ^c^	0.09 ^c^	N/A ^c^	0.97 ^c^	0.01 ^c^	N/A ^c^	N/A ^c^
rs4149056	0.16 ^a^	0.06 ^a^	0.15 ^a^	0.14 ^a^	0.14 ^a^	0.14 ^a^	0.02 ^a^	0.12 ^a^	0.02 ^a^	0.08 ^a^	0.21 ^a^	0.01 ^a^	0.02 ^a^
		6.7 ^b^	0.19 ^b^	0.59 ^b^	0.39 ^b^	0.28 ^b^	26.1 ^b^	1.61 ^b^	24.8 ^b^	5.1 ^b^	2.2 ^b^	32.6 ^b^	23.9 ^b^
		0.01 ^c^	0.65 ^c^	0.44 ^c^	0.52 ^c^	0.59 ^c^	33 × 10^−8 c^	0.20 ^c^	62 × 10^−8 c^	0.02 ^c^	0.13 ^c^	1 × 10^−8 c^	1 × 10^−6 c^
rs7294	0.33 ^a^	0.48 ^a^	0.31 ^a^	0.04 ^a^	0.17 ^a^	0.42 ^a^	0.67 ^a^	0.09 ^a^	0.43 ^a^	0.35 ^a^	0.34 ^a^	0.51 ^a^	0.46 ^a^
		6.9 ^b^	0.23 ^b^	N/A ^b^	15.7 ^b^	3.1 ^b^	N/A ^b^	N/A ^b^	4.2 ^b^	0.11 ^b^	2 × 10^−3 b^	15.6 ^b^	7.1 ^b^
		0.01 ^c^	0.62 ^c^	N/A ^c^	1 × 10^−4 c^	0.07 ^c^	N/A ^c^	N/A ^c^	0.03 ^c^	0.74 ^c^	0.96 ^c^	1 × 10^−4 c^	7 × 10^−3 c^
rs9934438	0.47 ^a^	0.15 ^a^	0.43 ^a^	0.96 ^a^	0.82 ^a^	0.36 ^a^	0.17 ^a^	0.90 ^a^	0.04 ^a^	0.47 ^a^	0.48 ^a^	0.03 ^a^	0.06 ^a^
		N/A ^b^	0.8 ^b^	N/A ^b^	N/A ^b^	5.8 ^b^	N/A ^b^	N/A ^b^	N/A ^b^	0.01 ^b^	0.01 ^b^	N/A ^b^	N/A ^b^
		N/A ^c^	0.35 ^c^	N/A ^c^	N/A ^c^	0.01 ^c^	N/A ^c^	N/A ^c^	N/A ^c^	0.93 ^c^	0.93 ^c^	N/A ^c^	N/A ^c^
rs12659	0.45 ^a^	0.40 ^a^	0.42 ^a^	0.49 ^a^	0.53 ^a^	0.39 ^a^	0.36 ^a^	0.54 ^a^	0.53 ^a^	0.34 ^a^	0.44 ^a^	0.48 ^a^	0.55 ^a^
		0.67 ^b^	0.33 ^b^	0.71 ^b^	3.2 ^b^	1.3 ^b^	3.2 ^b^	4.3 ^b^	3.1 ^b^	3.7 ^b^	0.002 ^b^	0.59 ^b^	4.9 ^b^
		0.41 ^c^	0.56 ^c^	0.39 ^c^	0.07 ^c^	0.24 ^c^	0.07 ^c^	0.03 ^c^	0.07 ^c^	0.05 ^c^	0.96 ^c^	0.44 ^c^	0.02 ^c^
rs1051266	0.42 ^a^	0.43 ^a^	0.57 ^a^	0.52 ^a^	0.46 ^a^	0.60 ^a^	0.60 ^a^	0.46 ^a^	0.31 ^a^	0.65 ^a^	0.55 ^a^	0.33 ^a^	0.31 ^a^
		6.7 ^b^	0.01 ^b^	1.2 ^b^	5.7 ^b^	0.21 ^b^	0.42 ^b^	6.6 ^b^	32.6 ^b^	1.8 ^b^	0.31 ^b^	29.2 ^b^	31.0 ^b^
		0.01 ^c^	0.89 ^c^	0.25 ^c^	0.01 ^c^	0.64 ^c^	0.51 ^c^	0.01 ^c^	1 × 10^−8 c^	0.17 ^c^	0.57 ^c^	6 × 10^−8 c^	3 × 10^−8 c^
rs1131596	0.42 ^a^	0.38 ^a^	0.57 ^a^	0.52 ^a^	0.46 ^a^	0.60 ^a^	0.61 ^a^	0.46 ^a^	0.24 ^a^	0.65 ^a^	0.55 ^a^	0.28 ^a^	0.28 ^a^
		0.72 ^b^	5.1 ^b^	1.5 ^b^	0.03 ^b^	7.8 ^b^	9.6 ^b^	0.09 ^b^	17.2 ^b^	11.8 ^b^	3.5 ^b^	10.8 ^b^	110.3 ^b^
		0.39 ^c^	0.02 ^c^	0.22 ^c^	0.87 ^c^	5 × 10^−3 c^	1 × 10^−3 c^	0.77 ^c^	3 × 10^−4 c^	0.001 ^c^	0.06 ^c^	1 × 10^−3 c^	1 × 10^−3 c^
rs890293	0.08 ^a^	0.16 ^a^	0.05 ^a^	0.04 ^a^	0.02 ^a^	0.05 ^a^	0.05 ^a^	0.02 ^a^	0.14 ^a^	0.03 ^a^	0.05 ^a^	0.15 ^a^	0.10 ^a^
		6.4 ^b^	1.4 ^b^	2.3 ^b^	8.3 ^b^	1.4 ^b^	1.1 ^b^	6.6 ^b^	4.1 ^b^	3.2 ^b^	1.3 ^b^	6.5 ^b^	0.9 ^b^
		0.01 ^c^	0.24 ^c^	0.12 ^C^	0.004 ^c^	0.23 ^c^	0.29 ^c^	0.01 ^c^	0.04 ^c^	0.07 ^c^	0.24 ^c^	0.01 ^c^	0.34 ^c^
rs10264272	0.0 ^a^	0.05 ^a^	0.00 ^a^	0.00 ^a^	0.00 ^a^	0.00 ^a^	0.00 ^a^	0.0 ^a^	0.24 ^a^	0.02 ^a^	0.01 ^a^	0.17 ^a^	0.11 ^a^
		12.8 ^b^	N/A ^b^	N/A ^b^	N/A ^b^	N/A ^b^	N/A ^b^	N/A ^b^	N/A ^b^	6.0 ^b^	1.2 ^b^	N/A ^b^	30.8 ^b^
		3 × 10^−4c^	N/A ^c^	N/A ^c^	N/A ^c^	N/A ^c^	N/A ^c^	N/A ^c^	N/A ^c^	0.01 ^c^	0.27 ^c^	N/A ^c^	3 × 10^−8 c^
rs776746	0.09 ^a^	0.68 ^a^	0.04 ^a^	0.31 ^a^	0.31 ^a^	0.05 ^a^	0.28 ^a^	0.25 ^a^	0.88 ^a^	0.23 ^a^	0.05 ^a^	0.83 ^a^	0.75 ^a^
		N/A ^b^	4.3 ^b^	N/A ^b^	N/A ^b^	1.9 ^b^	27.8 ^b^	22.8 ^b^	N/A ^b^	17.6 ^b^	2.6 ^b^	N/A ^b^	N/A ^b^
		N/A ^c^	0.04 ^c^	N/A ^c^	N/A ^c^	0.17 ^c^	13 × 10^−8 c^	2 × 10^−6 c^	N/A ^c^	3 × 10^−5 c^	0.11 ^c^	N/A ^c^	N/A ^c^
rs4986913	0.0 ^a^	0.0 ^a^	0.0 ^a^	0.0 ^a^	0.0 ^a^	0.0 ^a^	0.005 ^a^	0.0 ^a^	0.0 ^a^	0.0 ^a^	0.0 ^a^	0.0 ^a^	0.0 ^a^
		N/A ^b^	N/A ^b^	N/A ^b^	N/A ^b^	N/A ^b^	16.7 ^b^	N/A ^b^	N/A ^b^	N/A ^b^	N/A ^b^	N/A ^b^	N/A ^b^
		N/A ^c^	N/A ^c^	N/A ^c^	N/A ^c^	N/A ^c^	4 × 10^−5 c^	N/A ^c^	N/A ^c^	N/A ^c^	N/A ^c^	N/A ^c^	N/A ^c^
rs4986910	0.01^ a^	0.0 ^a^	0.01 ^a^	0.0 ^a^	0.0 ^a^	0.01 ^a^	0.0 ^a^	0.0 ^a^	0.0 ^a^	0.0 ^a^	0.0 ^a^	0.0 ^a^	0.0 ^a^
		0.1 ^b^	0.1 ^b^	2.4 ^b^	2.2 ^b^	0.01 ^b^	2.4 ^b^	2.4 ^b^	2.3 ^b^	1.5 ^b^	2.5 ^b^	2.5 ^b^	2.3 ^b^
		0.75 ^c^	0.75 ^c^	0.12 ^c^	0.14 ^c^	0.93 ^c^	0.12 ^c^	0.12 ^c^	0.13 ^c^	0.22 ^c^	0.11 ^c^	0.11 ^c^	0.13 ^c^
rs2740574	0.03 ^a^	0.67 ^a^	0.02 ^a^	0.0 ^a^	0.0 ^a^	0.03 ^a^	0.08 ^a^	0.0 ^a^	0.83 ^a^	0.07 ^a^	0.03 ^a^	0.76 ^a^	0.66 ^a^
		N/A ^b^	0.78 ^b^	5.7 ^b^	5.1 ^b^	0.11 ^b^	6.1 ^b^	5.8 ^b^	N/A ^b^	3.9 ^b^	0.002 ^b^	N/A ^b^	N/A ^b^
		N/A ^c^	0.38 ^c^	0.02 ^c^	0.02 ^c^	073 ^c^	0.01 ^c^	0.02 ^c^	N/A ^c^	0.05 ^c^	0.96 ^c^	N/A^ c^	N/A ^c^
rs4986893	0.004 ^a^	0.0 ^a^	0.0 ^a^	0.04 ^a^	0.07 ^a^	0.0 ^a^	0.0 ^a^	0.07 ^a^	0.01 ^a^	0.0 ^a^	0.0 ^a^	0.0 ^a^	5 × 10^−3 a^
		0.47 ^b^	0.74 ^b^	8.5 ^b^	16.7 ^b^	0.71 ^b^	0.02 ^b^	16.0 ^b^	196 ^b^	0.50 ^b^	0.83 ^b^	0.83 ^b^	0.04 ^b^
		0.48 ^c^	0.38 ^c^	3 × 10^−3 c^	4 × 10^−5 c^	0.39 ^c^	0.87 ^c^	6 × 10^−5 c^	0.42 ^c^	0.47 ^c^	0.35 ^c^	0.35 ^c^	8.3 × 10^−2 c^
rs4244285	0.08 ^a^	0.14 ^a^	0.13 ^a^	0.33 ^a^	0.26 ^a^	0.14 ^a^	0.33 ^a^	0.32 ^a^	0.21 ^a^	0.12 ^a^	0.09 ^a^	0.17 ^a^	0.15 ^a^
		3.4 ^b^	3.3 ^b^	N/A ^b^	27.7 ^b^	4.6 ^b^	N/A ^b^	N/A ^b^	16.7b	2.1 ^b^	0.32 ^b^	8.5 ^b^	5.8 ^b^
		0.06 ^c^	0.07 ^c^	N/A ^c^	14 × 10^−8 c^	0.03 ^c^	N/A ^c^	N/A ^c^	4 × 10^−5 c^	0.14 ^c^	0.57 ^c^	3 × 10^−3 c^	0.01 ^c^
rs1799853	0.12 ^a^	0.04 ^a^	0.15 ^a^	0.0 ^a^	0.0 ^a^	0.08 ^a^	0.05 ^a^	0.0 ^a^	0.0 ^a^	0.10 ^a^	0.15 ^a^	0.0 ^a^	0.03 ^a^
		5.7 ^b^	1.1 ^b^	25.8 ^b^	23.3 ^b^	0.97 ^b^	6.8 ^b^	26.0 ^b^	24.8 ^b^	0.21 ^b^	1.3 ^b^	27.0 ^b^	12.6 ^b^
		0.02 ^c^	0.28 ^c^	38 × 10^−8 c^	1 × 10^−6 c^	0.32 ^c^	9 × 10^−3 c^	33 × 10^−8 c^	62 × 10^−8 c^	0.64 ^c^	0.24 ^c^	2 × 10^−7 c^	3 × 10^−4 c^
rs28399454	0.0 ^a^	0.07 ^a^	0.0 ^a^	0.0 ^a^	0.0 ^a^	0.0 ^a^	0.0 ^a^	0.0 ^a^	0.06 ^a^	0.0 ^a^	0.0 ^a^	0.13 ^a^	0.15 ^a^
		19.3 ^b^	N/A ^b^	N/A ^b^	N/A ^b^	N/A ^b^	N/A ^b^	N/A ^b^	14.5 ^b^	N/A ^b^	N/A ^b^	N/A ^b^	N/A ^b^
		1 × 10^−5 c^	N/A ^c^	N/A ^c^	N/A ^c^	N/A ^c^	N/A ^c^	N/A ^c^	1× 10^−4 c^	N/A ^c^	N/A ^c^	N/A ^c^	N/A ^c^
rs1801272	0.0 ^a^	0.01 ^a^	0.04 ^a^	0.0 ^a^	0.0 ^a^	0.03 ^a^	0.01 ^a^	0.0 ^a^	0.0 ^a^	0.0 ^a^	0.05 ^a^	0.0 ^a^	0.0 ^a^
		2.1 ^b^	9.2 ^b^	N/A ^b^	N/A ^b^	7.1 ^b^	2.4 ^b^	N/A ^b^	N/A ^b^	N/A ^b^	12.2 ^b^	N/A ^b^	N/A ^b^
		0.14 ^c^	2 × 10^−3 c^	N/A ^c^	N/A ^c^	8 × 10^−3 c^	0.11 ^c^	N/A ^c^	N/A ^c^	N/A ^c^	47 × 10^−5 c^	N/A ^c^	N/A ^c^
rs28399433	0.07 ^a^	0.06 ^a^	0.05 ^a^	0.27 ^a^	0.18 ^a^	0.04 ^a^	0.19 ^a^	0.28 ^a^	0.09 ^a^	0.10 ^a^	0.06 ^a^	0.10 ^a^	0.06 ^a^
		1.4 ^b^	0.75 ^b^	33.2 ^b^	12.1 ^b^	1.3 ^b^	14.9 ^b^	N/A ^b^	0.64 ^b^	1.1 ^b^	0.04 ^b^	1.5 ^b^	0.1 ^b^
		0.23 ^c^	0.38 ^c^	1 × 10^−8 c^	5 × 10^−4 c^	0.25 ^c^	11 × 10^−5 c^	N/A ^c^	0.42 ^c^	0.28 ^c^	0.83 ^c^	0.22 ^c^	0.74 ^c^
rs3745274	0.2 ^a^	0.35 ^a^	0.28 ^a^	0.27 ^a^	0.33 ^a^	0.23 ^a^	0.23 ^a^	0.22 ^a^	0.36 ^a^	0.31 ^a^	0.30 ^a^	0.40 ^a^	0.38 ^a^
		9.3 ^b^	3.2 ^b^	1.4 ^b^	8.3 ^b^	0.4 ^b^	21.0 ^b^	0.19 ^b^	13.0 ^b^	5.3 ^b^	5.9 ^b^	21.4 ^b^	16.3 ^b^
		2 × 10^−2 c^	0.07 ^c^	0.23 ^c^	4 × 10^−3 c^	0.51 ^c^	45 × 10^−7 c^	0.66 ^c^	4 × 10^−3 c^	0.02 ^c^	0.01 ^c^	3 × 10^−6 c^	5 × 10^−5 c^
rs28399499	0.0 ^a^	0.1 ^a^	0.0 ^a^	0.0 ^a^	0.0 ^a^	0.0 ^a^	0.0 ^a^	0.0 ^a^	0.06 ^a^	0.08 ^a^	0.0 ^a^	0.12 ^a^	0.05 ^a^
		26.0 ^b^	N/A ^b^	N/A ^b^	N/A ^b^	N/A ^b^	N/A ^b^	N/A ^b^	15.9b	2.0 ^b^	N/A ^b^	31.2 ^b^	15.0 ^b^
		34 × 10^−8 c^	N/A ^c^	N/A ^c^	N/A ^c^	N/A ^c^	N/A ^c^	N/A ^c^	6 × 10^−5 c^	0.16 ^c^	N/A ^c^	2 × 10^−8 c^	1 × 10^−4 c^
rs59421388	0.0 ^a^	0.04 ^a^	0.0 ^a^	0.0 ^a^	0.0 ^a^	0.0 ^a^	0.0 ^a^	0.0 ^a^	0.09 ^a^	0.0 ^a^	0.0 ^a^	0.11 ^a^	0.08 ^a^
		10.6 ^b^	N/A ^b^	N/A ^b^	N/A ^b^	N/A ^b^	N/A ^b^	N/A ^b^	N/A ^b^	N/A ^b^	N/A ^b^	28.6 ^b^	23.5 ^b^
		1 × 10^−2 c^	N/A ^c^	N/A ^c^	N/A ^c^	N/A ^c^	N/A ^c^	N/A ^c^	N/A ^c^	N/A ^c^	N/A ^c^	9 × 10^−8 c^	1 × 10^−6 c^
rs28371725	0.15 ^a^	0.02 ^a^	0.12 ^a^	0.03 ^a^	0.08 ^a^	0.07 ^a^	0.15 ^a^	0.01 ^a^	0.03 ^a^	0.02 ^a^	0.14 ^a^	0.01 ^a^	0.05 ^a^
		14.9 ^b^	0.62 ^b^	16.6 ^b^	4.5 ^b^	5.8 ^b^	1 × 10^−3 b^	30.1 ^b^	17.4 ^b^	15.8 ^b^	0.004 ^b^	28.7 ^b^	11.7 ^b^
		1 × 10^−4 c^	0.43 ^c^	4 × 10^−5 c^	0.03 ^c^	0.02 ^c^	0.97 ^c^	4 × 10^−8 c^	3 × 10^−5 c^	7 × 10^−5 c^	0.94 ^c^	8 × 10^−8 c^	6 × 10^−4 c^
rs61736512	0.0 ^a^	0.04 ^a^	0.0 ^a^	0.0 ^a^	0.0 ^a^	0.0 ^a^	0.0 ^a^	0.0 ^a^	0.17 ^a^	0.0 ^a^	0.0 ^a^	0.11 ^a^	0.09 ^a^
		10.6 ^b^	N/A ^b^	N/A ^b^	N/A ^b^	N/A ^b^	N/A ^b^	N/A ^b^	N/A ^b^	N/A ^b^	N/A ^b^	29.9 ^b^	25.0 ^b^
		1 × 10^−3 c^	N/A ^c^	N/A ^c^	N/A ^c^	N/A ^c^	N/A ^c^	N/A ^c^	N/A ^c^	N/A ^c^	N/A ^c^	4 × 10- ^c^	57 × 10^−8 c^
rs28371706	0.01 ^a^	0.15 ^a^	0.0 ^a^	0.0 ^a^	0.0 ^a^	0.0 ^a^	0.0 ^a^	0.0 ^a^	0.19 ^a^	0.0 ^a^	0.0 ^a^	0.25 ^a^	0.20 ^a^
		32.1 ^b^	1.5 ^b^	1.6 ^b^	1.4 ^b^	1.4 ^b^	1.6 ^b^	1.6 ^b^	N/A ^b^	1.0 ^b^	1.7 ^b^	N/A ^b^	N/A ^b^
		1 × 10^−1 c^	0.21 ^c^	0.20 ^c^	0.22 ^c^	0.23 ^c^	0.20 ^c^	0.20 ^c^	N/A ^c^	0.32 ^c^	0.19 ^c^	N/A ^c^	N/A ^c^
rs5030656	0.0 ^a^	0.01 ^a^	0.02 ^a^	0.0 ^a^	0.0 ^a^	0.04 ^a^	0.0 ^a^	0.0 ^a^	0.0 ^a^	0.01 ^a^	0.01 ^a^	0.0 ^a^	0.0 ^a^
		2.1 ^b^	5.2 ^b^	N/A ^b^	N/A ^b^	10.0 ^b^	N/A ^b^	N/A ^b^	N/A ^b^	2.0 ^b^	8.5 ^b^	N/A ^b^	N/A ^b^
		0.14 ^c^	0.02 ^c^	N/A ^c^	N/A ^c^	0.001 ^c^	N/A ^c^	N/A ^c^	N/A ^c^	0.16 ^c^	3 × 10^−3 c^	N/A ^c^	N/A ^c^
rs4124874	0.46 ^a^	0.22 ^a^	0.57 ^a^	0.72 ^a^	0.55 ^a^	0.62 ^a^	0.39 ^a^	0.69 ^a^	0.11 ^a^	0.48 ^a^	0.56 ^a^	0.09 ^a^	0.14 ^a^
		N/A ^b^	4.8 ^b^	33.5 ^b^	3.2 ^b^	11.0 ^b^	2.2 ^b^	24.0 ^b^	N/A ^b^	0.2 ^b^	4.2 ^b^	N/A ^b^	34.0 ^b^
		N/A ^c^	0.03 ^c^	1 × 10^−8 c^	0.07 ^c^	1 × 10^−2 c^	0.14 ^c^	99 × 10^−8 c^	N/A ^c^	0.67 ^c^	0.04 ^c^	N/A ^c^	1 × 10^−8 c^
rs10929302	0.39 ^a^	0.33 ^a^	0.30 ^a^	0.11 ^a^	0.15 ^a^	0.23 ^a^	0.44 ^a^	0.18 ^a^	0.36 ^a^	0.34 ^a^	0.24 ^a^	0.64 ^a^	0.30 ^a^
		1.4 ^b^	3.4 ^b^	N/A ^b^	30.4 ^b^	12.6 ^b^	0.9 ^b^	25.2 ^b^	0.5 ^b^	0.8 ^b^	12.5 ^b^	0.5 ^b^	3.4 ^b^
		0.32 ^c^	0.07 ^c^	N/A ^c^	3 × 10^−8 c^	4 × 10^−4 c^	0.32 ^c^	52 × 10^−8 c^	0.47 ^c^	0.36 ^c^	4 × 10^−4 c^	0.49 ^c^	0.06 ^c^
rs4148323	0.03 ^a^	0.01 ^a^	0.0 ^a^	0.23 ^a^	0.10 ^a^	0.0 ^a^	0.02 ^a^	0.13 ^a^	0.0 ^a^	0.02 ^a^	0.0 ^a^	0.0 ^a^	0.0 ^a^
		1.4 ^b^	5.5 ^b^	N/A ^b^	11.0 ^b^	5.0 ^b^	0.29 ^b^	18.0 ^b^	5.5 ^b^	0.04 ^b^	5.9 ^b^	5.9 ^b^	5.2 ^b^
		0.23	0.02 ^c^	N/A ^c^	9 × 10^−4 c^	0.02 ^c^	0.59 ^c^	2 × 10^−5 c^	0.02 ^c^	0.83 ^c^	0.01 ^c^	0.01 ^c^	0.02 ^c^
rs4680	0.41 ^a^	0.27 ^a^	0.46 ^a^	0.31 ^a^	0.26 ^a^	0.52 ^a^	0.43 ^a^	0.28 ^a^	0.28 ^a^	0.39 ^a^	0.45 ^a^	0.30 ^a^	0.36 ^a^
		7.4 ^b^	1.1 ^b^	8.3 ^b^	10.1 ^b^	5.4 ^b^	0.2 ^b^	8.6 ^b^	7.8 ^b^	0.1 ^b^	0.7 ^b^	6.0 ^b^	3.9 ^b^
		7 × 10^−3 c^	0.29 ^c^	4 × 10^−3 c^	15 × 10^−4 c^	0.02 ^c^	0.63 ^c^	0.00 ^c^	5 × 10^−3 c^	0.75 ^c^	0.40 ^c^	0.01 ^c^	0.04 ^c^
rs1695	0.33 ^a^	0.30 ^a^	0.45 ^a^	0.39 ^a^	0.18 ^a^	0.22 ^a^	0.31 ^a^	0.31 ^a^	0.10 ^a^	0.51 ^a^	0.56 ^a^	0.29 ^a^	0.39 ^a^
		32.8 ^b^	6.0 ^b^	2.0 ^b^	12.4 ^b^	6.3 ^b^	0.1 ^b^	0.2 ^b^	N/A ^b^	15.1 ^b^	19.2 ^b^	0.7 ^b^	2.4 ^b^
		1 × 10^−8 c^	0.01 ^c^	0.15 ^c^	4 × 10^−4 c^	0.01 ^c^	0.81 ^c^	0.66 ^c^	N/A ^c^	1 × 10^−4 c^	1 × 10^−5 c^	0.41 ^c^	0.12 ^c^
rs1138272	0.16 ^a^	0.10 ^a^	0.02 ^a^	0.09 ^a^	0.00 ^a^	0.00 ^a^	0.05 ^a^	0.08 ^a^	0.00 ^a^	0.01 ^a^	0.05 ^a^	0.05 ^a^	0.00 ^a^
		4.7 ^b^	14.0 ^b^	3.5 ^a^	N/A ^b^	31.6 ^b^	10.6 ^b^	5.6 ^b^	N/A ^b^	25.7b	8.1 ^b^	13.0 ^b^	N/A ^b^
		0.03 ^c^	2 × 10^−4 c^	0.06 ^c^	N/A ^c^	2 × 10^−8 c^	1 × 10^−3 c^	0.02 ^c^	N/A ^c^	41 × 10^−8 c^	5 × 10^−3 c^	3 × 10^−4 c^	N/A ^c^

CAR: Circassian living in Jordan (the current study), ASW: African ancestry in Southwest USA, CEU: Utah, USA residents with Northern and Western European ancestry from the CEPH collection, CHB: Han Chinese in Beijing, China, CDX: Chinese Dai in Xishuangbanna, China, GIH: Gujarati Indians in Houston, Texas, USA, GBR: British in England and Scotland, JPT: Japanese in Tokyo, Japan, LWK: Luhya in Webuye, Kenya, MXL: Mexican ancestry in Los Angeles, California, USA, TSI: Toscani in Italy, YRI: Yoruba in Ibadan, Nigeria, ACB: African Caribbean in Barbados. N/A: Not Applicable. a: minor allele frequency, b: chi-square value, c: *p*-value, when *p*-value < 0.05 is considered a significant difference.

**Table 4:** Significant variants within the pharmacogenes in Circassian compared to six ExAC populations worldwide.

SNP\Population	CAR	African	East Asian	Latino	European (Non Finnish)	South Asian	European (Finnish)
rs1065776	0.02 ^a^	0.18 ^a^	0.04 ^a^	0.05 ^a^	0.04 ^a^	0.11 ^a^	0.07 ^a^
		N/A ^b^	3.6 ^b^	5.7 ^b^	3.1 ^b^	22.1 ^b^	9.4 ^b^
		N/A ^c^	0.06 ^c^	0.02 ^c^	0.08 ^c^	3 × 10^−6 c^	2 × 10^−3 c^
rs701265	0.14 ^a^	0.69 ^a^	0.29 ^a^	0.22 ^a^	0.14 ^a^	0.19 ^a^	0.15 ^a^
		N/A ^b^	27.1 ^b^	10.8 ^b^	0.1 ^b^	4.6 ^b^	0.21 ^b^
		N/A ^c^	2 × 10^−7 c^	1 × 10^−3 c^	0.80 ^c^	0.03 ^c^	0.64 ^c^
rs2066702	0.03 ^a^	0.19 ^a^	1 × 10^−4 a^	0.01 ^a^	2 × 10^−3 a^	2 × 10^−3 a^	0.00 ^a^
		N/A ^b^	N/A ^b^	1.4 ^b^	N/A ^b^	N/A ^b^	N/A ^b^
		N/A ^c^	N/A ^c^	0.22 ^c^	N/A ^c^	N/A ^c^	N/A ^c^
rs1229984	0.13 ^a^	0.99 ^a^	0.27 ^a^	0.94 ^a^	0.95 ^a^	0.95 ^a^	0.99 ^a^
		N/A ^b^	25.4 ^b^	N/A ^b^	N/A ^b^	N/A ^b^	N/A ^b^
		N/A ^c^	45 × 10^−8 c^	N/A ^c^	N/A ^c^	N/A ^c^	N/A ^c^
rs698	0.25 ^a^	0.15 ^a^	0.08 ^a^	0.33 ^a^	0.40 ^a^	0.32 ^a^	0.51 ^a^
		21.1 ^b^	N/A ^b^	6.9 ^b^	21.9 ^b^	5.9 ^b^	N/A ^b^
		4 × 10^−6 c^	N/A ^c^	0.01 ^c^	3 × 10^−6 c^	0.01 ^c^	N/A ^c^
rs3846662	0.48 ^a^	0.88 ^a^	0.53 ^a^	0.47 ^a^	0.44 ^a^	0.59 ^a^	0.47 ^a^
		N/A ^b^	2.8 ^b^	0.13 ^b^	1.3 ^b^	11.5 ^b^	0.1 ^b^
		N/A ^c^	0.09 ^c^	0.71 ^c^	0.24 ^c^	1 × 10^−3 c^	0.75 ^c^
rs1042713	0.46 ^a^	0.49 ^a^	0.55 ^a^	0.42 ^a^	0.38 ^a^	0.46 ^a^	0.13 ^a^
		1.2 ^b^	8.2 ^b^	1.4 ^b^	6.4 ^b^	3 × 10^−3 b^	N/A ^b^
		0.25 ^c^	4 × 10^−3 c^	0.22 ^c^	0.01 ^c^	0.95 ^c^	0.70 ^c^
rs1042714	0.32 ^a^	0.82 ^a^	0.91 ^a^	0.83 ^a^	0.58 ^a^	0.79 ^a^	0.63 ^a^
		N/A ^b^	N/A ^b^	N/A ^b^	N/A ^b^	N/A ^b^	N/A ^b^
		N/A ^c^	N/A ^c^	N/A ^c^	N/A ^c^	N/A ^c^	N/A ^c^
rs2066853	0.16 ^a^	0.45 ^a^	0.37 ^a^	0.12 ^a^	0.10 ^a^	0.14 ^a^	0.11 ^a^
		N/A ^b^	N/A ^b^	3.3 ^b^	10.3 ^b^	1.0 ^b^	4.9 ^b^
		N/A ^c^	N/A ^c^	0.06 ^c^	1 × 10^−3 c^	0.30 ^c^	0.02 ^c^
rs3815459	0.27 ^a^	0.37 ^a^	0.72 ^a^	0.38 ^a^	0.33 ^a^	0.39 ^a^	0.44 ^a^
		7.9 ^b^	N/A ^b^	5.0 ^b^	3.8 ^b^	15.6 ^b^	2.1 ^b^
		4 × 10^−3 c^	N/A ^c^	0.02 ^c^	0.05 ^c^	1 × 10^−4 c^	0.14 ^c^
rs5219	0.41 ^a^	0.94 ^a^	0.64 ^a^	0.61 ^a^	0.63 ^a^	0.63 ^a^	0.53 ^a^
		N/A ^b^	N/A ^b^	N/A ^b^	N/A ^b^	N/A ^b^	14.0 ^b^
		N/A ^c^	N/A ^c^	N/A ^c^	N/A ^c^	N/A ^c^	2 × 10^−4 c^
rs4149056	0.16 ^a^	0.03 ^a^	0.13 ^a^	0.11 ^a^	0.16 ^a^	0.05 ^a^	4.1 ^a^
		N/A ^b^	2.8 ^b^	8.0 ^b^	3 × 10^−3 b^	N/A ^b^	N/A ^b^
		N/A ^c^	0.09 ^c^	4 × 10^−3 c^	0.95 ^c^	N/A ^c^	0.04 ^c^
rs12659	0.45 ^a^	0.55 ^a^	0.49 ^a^	0.56 ^a’^	0.58 ^a^	0.64 ^a^	0.56 ^a^
		10.2 ^b^	1.8 ^b^	14.7 ^b^	19.6 ^b^	N/A ^b^	N/A ^b^
		1 × 10^−3 c^	0.17 ^c^	1 × 10^−4 c^	1 × 10^−5 c^	N/A ^c^	N/A ^c^
rs1051266	0.42 ^a^	0.42 ^a^	0.52 ^a^	0.59 ^a^	0.60 ^a^	0.61 ^a^	0.60 ^a^
		0.1 ^b^	8.6 ^b^	29.1 ^b^	32.2 ^b^	N/A ^b^	32.5 ^b^
		0.80 ^c^	3 × 10^−3 c^	7 × 10^−8 c^	1 × 10^−8 c^	N/A ^c^	1 × 10^−8 c^
rs1131596	0.42 ^a^	0.36 ^a^	0.51 ^a^	0.56 ^a^	0.59 ^a^	0.58 ^a^	0.61 ^a^
		4.7 ^b^	5.9 ^b^	15.2 ^b^	30.5 ^b^	23.3 ^b^	20.2 ^b^
		0.03 ^c^	0.01 ^c^	1 × 10^−4 c^	3 × 10^−8 c^	1 × 10^−5 c^	1 × 10^−5 c^
rs10264272	0.0 ^a^	0.12 ^a^	0.0 ^a^	0.01 ^a^	1 × 10^−3 a^	2 × 10^−4 a^	0.0 ^a^
		N/A ^b^	N/A ^b^	2.17 ^b^	0.21 ^b^	0.06 ^b^	N/A ^b^
		N/A ^c^	N/A ^c^	0.15 ^c^	0.64 ^c^	0.80 ^c^	N/A ^c^
rs4986913	0.0 ^a^	0.0 ^a^	0.0 ^a^	0.0 ^a^	0.0 ^a^	1× 10^−3 a^	0.0 ^a^
		N/A ^b^	N/A ^b^	N/A ^b^	N/A ^b^	0.20 ^b^	N/A ^b^
		N/A ^c^	N/A ^c^	N/A ^c^	N/A ^c^	0.65 ^c^	N/A ^c^
rs4986910	0.01 ^a^	1 × 10^−3 a^	0.0 ^a^	2 × 10^−3 a^	7 × 10^−3 a^	0.0 ^a^	0.02 ^a^
		18.2 ^b^	N/A ^b^	12.1 ^b^	0.6 ^b^	N/A ^b^	0.9 ^b^
		2 × 10^−5 c^	N/A ^c^	5 × 10^−4 c^	0.43 ^c^	N/A ^c^	0.32 ^c^
rs4986909	0.0 ^a^	0.0 ^a^	0.0 ^a^	2 × 10^−3 a^	5.99 × 10^−5^	0.0 ^a^	0.0 ^a^
		N/A ^b^	N/A ^b^	0.04 ^b^	0.01 ^b^	N/A ^b^	N/A ^b^
		N/A ^c^	N/A ^c^	0.83 ^c^	0.90 ^c^	N/A ^c^	N/A ^c^
rs12721634	0.0 ^a^	N/A	N/A	N/A	N/A	N/A	N/A
rs4986893	0.004 ^a^	5 × 10^−4 a^	0.07 ^a^	4 × 10^−4 a^	2 × 10^−4 a^	4 × 10^−3 a^	2 × 10^−4 a^
		5.1 ^b^	16.3 ^b^	9.7 ^b^	18.2 ^b^	0.01 ^b^	11.9 ^b^
		0.02 ^c^	5 × 10 ^c^	1 × 10^−3 c^	2 × 10^−5 c^	0.94 ^c^	5.5 × 10^−4 c^
rs4244285	0.08 ^a^	0.18 ^a^	0.31 ^a^	0.10 ^a^	0.15 ^a^	0.34 ^a^	0.18 ^a^
		17.6 ^b^	N/A ^b^	1.3 ^b^	9.5 ^b^	N/A ^b^	18.0 ^b^
		3 × 10^−5 c^	N/A ^c^	0.24 ^c^	2 × 10^−3 c^	N/A ^c^	22 × 10^−6 c^
rs1799853	0.12 ^a^	0.02 ^a^	3 × 10^−4 a^	0.07 ^a^	0.13 ^a^	0.05 ^a^	0.12 ^a^
		N/A ^b^	N/A ^b^	10.4 ^b^	0.21 ^b^	28.4 ^b^	N/A ^b^
		N/A ^c^	N/A ^c^	1 × 10^−3 c^	0.64 ^c^	1 × 10^−7 c^	N/A ^c^
rs28399454	0.0 ^a^	0.11 ^a^	0.0 ^a^	6 × 10^−3 a^	4 × 10^−4 a^	0.0 ^a^	0.0 ^a^
		32.1 ^b^	N/A ^b^	1.5 ^b^	0.1 ^b^	N/A ^b^	N/A ^b^
		1 × 10^−8 c^	N/A ^c^	0.21 ^c^	0.76 ^c^	N/A ^c^	N/A ^c^
rs1801272	0.0 ^a^	5 × 10^−3 a^	0.0 ^a^	0.012 ^a^	0.03 ^a^	1.1 × 10^−2 a^	2.3 × 10^−2 a^
		1.2 ^b^	N/A ^b^	3.1 ^b^	6.7 ^b^	3.0 ^b^	6.1 ^b^
		0.28 ^c^	N/A ^c^	0.08 ^c^	9 × 10^−3 c^	0.08 ^c^	0.01 ^c^
rs28399433	0.07 ^a^	8.3 × 10^−2 a^	0.23 ^a^	13.8 × 10^−2 a^	6.8 × 10^−2 a^	14.4 × 10^−2 a^	0.11 ^a^
		0.55 ^b^	N/A ^b^	9.7 ^b^	0.01 ^b^	11.2 ^b^	4.2 ^b^
		0.45 ^c^	N/A ^c^	2 × 10^−3 c^	0.90 ^c^	8 × 10^−4 c^	0.04 ^c^
rs3745274	0.2 ^a^	0.37 ^a^	0.19 ^a^	0.32 ^a^	0.24 ^a^	0.19 ^a^	0.19 ^a^
		26.9 ^b^	0.41 ^b^	14.5 ^b^	1.8 ^b^	N/A ^b^	0.28 ^b^
		21 × 10^−8 c^	0.52 ^c^	1 × 10^−4 c^	0.18 ^c^	N/A ^c^	0.60 ^c^
rs28399499	0.0 ^a^	0.07 ^a^	0.0 ^a^	3 × 10^−3 a^	1 × 10^−4 a^	1 × 10^−4 a^	0.0 ^a^
		19.4 ^b^	N/A ^b^	0.83 ^b^	0.04 ^b^	0.04 ^b^	N/A ^b^
		1 × 10^−5 c^	N/A ^c^	0.36 ^c^	0.85 ^c^	0.85 ^c^	N/A ^c^
rs59421388	0.0 ^a^	9.2 × 10^−2 a^	1 × 10^−4 a^	4 × 10^−3 a^	3 × 10^−4 a^	1 × 10^−4 a^	0.0 ^a^
		26.0 ^b^	0.03 ^b^	0.96 ^b^	0.1 ^b^	0.03 ^b^	N/A ^b^
		35 × 10^−8 c^	0.86 ^c^	0.33 ^c^	0.79 ^c^	0.86 ^c^	N/A ^c^
rs28371725	0.15 ^a^	0.03 ^a^	0.03 ^a^	0.03 ^a^	0.09 ^a^	0.15 ^a^	0.03 ^a^
		N/A ^b^	N/A ^b^	N/A ^b^	8.0 ^b^	0.27 ^b^	N/A ^b^
		N/A ^c^	N/A ^c^	N/A ^c^	5 × 10^−3 c^	0.60 ^c^	N/A ^c^
rs61736512	0.0 ^a^	0.10 ^a^	5 × 10^−4 a^	4 × 10^−3 a^	3 × 10^−4 a^	2 × 10^−4 a^	0.0 ^a^
		28.5 ^b^	0.1 ^a^	1.0 ^a^	0.07 ^a^	0.06 ^a^	N/A ^b^
		9 × 10^−8 c^	0.73 ^c^	0.32 ^c^	0.79 ^c^	0.80 ^c^	N/A ^c^
rs28371706	0.01 ^a^	0.197 ^a^	0.0 ^a^	7 × 10^−3 a^	2 × 10^−3 a^	1 × 10^−3 a^	2 × 10^−4 a^
		N/A ^b^	N/A ^b^	0.01 ^b^	3.7 ^b^	N/A ^b^	31.2 ^b^
		N/A ^c^	N/A ^c^	0.90 ^c^	0.05 ^c^	N/A ^c^	2 × 10^−8 c^
rs5030656	0.0 ^a^	04 × 10^−3 a^	0.0 ^a^	1.2 × 10^−2 a^	0.03 ^a^	2 × 10^−3 a^	1.5 × 10^−2 a^
		1.1 ^b^	N/A ^b^	3.3 ^b^	8.0 ^b^	0.52 ^b^	4.0 ^b^
		0.28 ^c^	N/A ^c^	0.07 ^c^	4 × 10^−3 c^	0.47 ^c^	0.04 ^c^
rs4148323	0.03 ^a^	9 × 10^−4 a^	0.15 ^a^	0.03 ^a^	4 × 10^−3 a^	0.02 ^a^	0.05 ^a^
		N/A ^b^	31.0 ^b^	0.01 ^b^	N/A ^b^	0.9 ^b^	2.0 ^b^
		N/A ^c^	3 × 10^−8 c^	0.90 ^c^	N/A ^c^	0.34 ^c^	0.51 ^c^
rs4680	0.41 ^a^	0.32 ^a^	0.28 ^a^	0.41 ^a^	0.53 ^a^	0.45 ^a^	0.57 ^a^
		10.4 ^b^	20.5 ^b^	0.1 ^b^	13.3 ^b^	1.1 ^b^	25.0 ^b^
		1 × 10^−3 c^	6 × 10^−6 c^	0.82 ^c^	3 × 10^−4 c^	0.28 ^c^	57 × 10^−8 c^
rs1695	0.33 ^a^	0.44 ^a^	0.18 ^a^	0.53 ^a^	0.31 ^a^	0.29 ^a^	0.27 ^a^
		13.4 ^b^	N/A ^b^	N/A ^b^	0.1 ^b^	0.2 ^b^	3.7 ^b^
		2 × 10^−4 c^	N/A ^c^	N/A ^c^	0.72 ^c^	0.15 ^c^	0.05 ^c^
rs1138272	0.16 ^a^	0.02 ^a^	3 × 10^−4 a^	0.03 ^a^	0.08 ^a^	0.07 ^a^	0.08 ^a^
		N/A ^b^	N/A ^b^	N/A ^b^	21.0 ^b^	28.3 ^b^	16.1 ^b^
		N/A ^c^	N/A ^c^	N/A ^c^	4 × 10^−6 c^	1 × 10^−7 c^	6 × 10^−5 c^

CAR: Circassian living in Jordan (the current study). N/A: Not Applicable. a: minor allele frequency, b: chi-square value, c: *p*-value, when *p*-value < 0.05 is considered a significant difference.

## References

[1] Engen R.M., Marsh S., Van Booven D.J., McLeod H.L. (2006). Ethnic differences in pharmacogenetically relevant genes. Curr. Drug Targets.

[2] Kruglyak L., Nickerson D.A. (2001). Variation is the spice of life. Nat. Genet..

[3] Tishkoff S.A., Kidd K.K. (2004). Implications of biogeography of human populations for ‘race’ and medicine. Nat. Genet..

[4] Mamotte C.D. (2006). Genotyping of single nucleotide substitutions. Clin. Biochem. Rev..

[5] Peters E.J., McLeod H.L. (2008). Ability of whole-genome SNP arrays to capture ‘must have’ pharmacogenomic variants. Pharmacogenomics.

[6] Wang L., Aikemu A., Yibulayin A., Du S., Geng T., Wang B., Zhang Y., Jin T., Yang J. (2015). Genetic polymorphisms of pharmacogenomic VIP variants in the Uygur population from northwestern China. BMC Genet..

[7] Zhang J., Jin T., Yunus Z., Li X., Geng T., Wang H., Cui Y., Chao C. (2014). Genetic polymorphisms of VIP variants in the Tajik ethnic group of northwest China. BMC Genet..

[8] Benet L.Z., Kroetz D., Sheiner L., Hardman J., Limbird L. (1999). Pharmacokinetics: The dynamics of drug absorption, distribution, metabolism, and elimination. Goodman and Gilman’s the Pharmacological Basis of Therapeutics.

[9] He Y., Yang H., Geng T., Feng T., Yuan D., Kang L., Luo M., Jin T. (2015). Genetic polymorphisms of pharmacogenomic VIP variants in the lhoba population of southwest China. Int. J. Clin. Exp. Pathol..

[10] Serre D., Pääbo S. (2004). Evidence for gradients of human genetic diversity within and among continents. Genome Res..

[11] Johnson J.A. (2008). Ethnic differences in cardiovascular drug response: Potential contribution of pharmacogenetics. Circulation.

[12] Kristiansson K., Naukkarinen J., Peltonen L. (2008). Isolated populations and complex disease gene identification. Genome Biol..

[13] Dajani R., Fathallah R., Arafat A., AbdulQader M.E., Hakooz N., Al-Motassem Y., El-Khateeb M. (2013). Prevalence of MTHFR C677T single nucleotide polymorphism in genetically isolated populations in Jordan. Biochem. Genet..

[14] Shastry B. (2005). Pharmacogenetics and the concept of individualized medicine. Pharm. J..

[15] Risch N., Burchard E., Ziv E., Tang H. (2002). Categorization of humans in biomedical research: Genes, race and disease. Genome Biol..

[16] Evans W.E., Johnson J.A. (2001). Pharmacogenomics: The Inherited Basis for Interindividual Differences in Drug Response. Annu. Rev. Genom. Hum. Genet..

[17] Lynch T., Price A. (2007). The effect of cytochrome P450 metabolism on drug response, interactions, and adverse effects. Am. Fam. Physician.

[18] Ingelman-Sundberg M. (2004). Pharmacogenetics of cytochrome P450 and its applications in drug therapy: The past, present and future. Trends Pharm. Sci..

[19] Jancova P., Anzenbacher P., Anzenbacherova E., Jancova P., Anzenbacher P., Anzenbacherova E. (2010). Phase II drug metabolizing enzymes. Biomed. Pap. Med. Fac. Univ. Palacky Olomouc Czech. Repub..

[20] Cashman J.R., Perotti B.Y., Berkman C.E., Lin J. (1996). Pharmacokinetics and molecular detoxication. Environ. Health Perspect..

[21] Iyanagi T. (2007). Molecular mechanism of phase I and phase II drug-metabolizing enzymes: Implications for detoxification. Int. Rev. Cytol..

[22] Vatansever S., Tekin F., Salman E., Altintoprak E., Coskunol H., Akarca U.S. (2015). Genetic polymorphisms of ADH1B, ADH1C and ALDH2 in Turkish alcoholics: Lack of association with alcoholism and alcoholic cirrhosis. Bosn. J. Basic Med. Sci..

[23] Yee S.W., Gong L., Badagnani I., Giacomini K.M., Klein T.E., Altman R.B. (2010). SLC19A1 pharmacogenomics summary. Pharm. Genom..

[24] Heutink P., Oostra B.A. (2002). Gene finding in genetically isolated populations. Hum. Mol. Genet..

[25] Zeggini E. (2014). Using genetically isolated populations to understand the genomic basis of disease. Genome Med..

[26] Newton-Cheh C., Guo C.Y., Larson M.G., Musone S.L., Surti A., Camargo A.L., Drake J.A., Benjamin E.J., Levy D., D’Agostino R.B. (2007). Common Genetic Variation in KCNH2 Is Associated with QT Interval Duration. Circulation.

[27] de Jonge R., Hooijberg J.H., van Zelst B.D., Jansen G., van Zantwijk C.H., Kaspers G.J., Peters G.J., Ravindranath Y., Pieters R., Lindemans J. (2005). Effect of polymorphisms in folate-related genes on in vitro methotrexate sensitivity in pediatric acute lymphoblastic leukemia. Blood.

[28] Sangkuhl K., Berlin D.S., Altman R.B., Klein T.E., Berlin D., Altman R.B., Klein T. (2009). PharmGKB: Understanding the effects of individual genetic variants. Drug Metab..

[29] Genvigir F.D., Nishikawa A.M., Felipe C.R., Tedesco-Silva H.J.R., Oliveira N., Salazar A.B.C., Medina-Pestana J.O., Doi S.Q., Hirata M.H., Hirata R.D.C. (2017). Influence of ABCC2, CYP2C8, and CYP2J2 Polymorphisms on Tacrolimus and Mycophenolate Sodium-Based Treatment in Brazilian Kidney Transplant Recipients. Pharmacotherapy.

[30] Ngamjanyaporn P., Thakkinstian A., Verasertniyom O., Chatchaipun P., Vanichapuntu M., Nantiruj K., Totemchokchyakarn K., Attia J., Janwityanujit S. (2011). Pharmacogenetics of cyclophosphamide and CYP2C19 polymorphism in Thai systemic lupus erythematosus. Rheumatol. Int..

[31] Kunugi H., Nanko S., Ueki A., Otsuka E., Hattori M., Hoda F., Vallada H.P., Arranz M.J., Collier D.A. (1997). High and low activity alleles of catechol-O-methyltransferase gene: Ethnic difference and possible association with Parkinson’s disease. Neurosci. Lett..

[32] Yoritaka A., Hattori N., Yoshino H., Mizuno Y. (1997). Catechol-Omethyltransferase genotype and susceptibility to Parkinson’s disease in Japan. J. Neural Transm..

[33] AL-Eitan L., Haddad Y. (2014). Emergence of pharmacogenomics in academic medicine and public health in Jordan: History, present state and prospects. Curr. Pharmacogenom. Person. Med..

[34] AL-Eitan L., Tarkhan A. (2014). Practical challenges and translational issues in pharmacogenomics and personalized medicine from 2010 onwards. Curr. Pharmacogenom. Person. Med..

[35] AL-Eitan L., Al-Dalalah I., Elshammari A., Khreisat W., Almasri A. (2018). The impact of potassium channel gene polymorphisms on antiepileptic drug responsiveness in Arab patients with epilepsy. J. Pers. Med..

[36] Al-Eitan L., Almasri A., Khasawneh R. (2018). Impact of CYP2C9 and VKORC1 polymorphisms on warfarin sensitivity and responsiveness in Jordanian cardiovascular patients during the initiation therapy. Genes.

[37] Al-Eitan L., Almasri A., Al-Habahbeh S. (2019). Effects of coagulation factor VII polymorphisms on warfarin sensitivity and responsiveness in Jordanian cardiovascular patients during the initiation and maintenance phases of warfarin therapy. Pharmgenom. Pers. Med..

[38] Al-Eitan L., Almasri A., Al-Habahbeh S. (2019). Impact of a variable number tandem repeat in the CYP2C9 promoter on warfarin sensitivity and responsiveness in Jordanians with cardiovascular disease. Pharmgenom. Pers. Med..

[39] Al-Eitan L., Almomani B., Nassar A., Elsaqa B., Saadeh N. (2019). Metformin pharmacogenetics: Effects of SLC22A1, SLC22A2, and SLC22A3 polymorphisms on glycemic control and HbA1c levels. J. Pers. Med..

[40] AL-Eitan L., Al-Dalalah I., Aljamal H. (2019). Effects of GRM4, SCN2A and SCN3B polymorphisms on antiepileptic drugs responsiveness and epilepsy susceptibility. Saudi Pharm. J..

[41] Al-Eitan L., Almasri A., Khasawneh R. (2019). Effects of CYP2C9 and VKORC1 polymorphisms on warfarin sensitivity and responsiveness during the stabilization phase of therapy. Saudi Pharm. J..

[42] AL-Eitan L., Al-Dalalah I., Mustafa M., Alghamdi M., Elshammari A., Khreisat W., Aljamal H. (2019). Effects of MTHFR and ABCC2 gene polymorphisms on antiepileptic drug responsiveness in Jordanian epileptic patients. Pharmgenom. Pers. Med..

[43] Al-Eitan L., Rababa’h D., Alghamdi M., Khasawneh R. (2019). Role of four ABC transporter genes in pharmacogenetic susceptibility to breast cancer in Jordanian patients. J. Oncol..

[44] Al-Eitan L., Mohammad N., Al-Maqableh H., Hakooz N., Dajani R. (2019). Genetic polymorphisms of pharmacogenomic VIP variants in the Circassian subpopulation from Jordan. Curr. Drug Metab..

